# Editorial: Awake functional imaging of small animals

**DOI:** 10.3389/fnins.2023.1226623

**Published:** 2023-06-13

**Authors:** Mehdi Behroozi, Onur Güntürkün, Annemie Van der Linden

**Affiliations:** ^1^Department of Biopsychology, Faculty of Psychology, Institute of Cognitive Neuroscience, Ruhr-University Bochum, Bochum, Germany; ^2^Research Center One Health Ruhr, Research Alliance Ruhr, Ruhr-University Bochum, Bochum, Germany; ^3^Bio-Imaging Lab, University of Antwerp, Antwerp, Belgium; ^4^μNEURO Research Centre of Excellence, University of Antwerp, Antwerp, Belgium

**Keywords:** fMRI, PET, functional ultrasound, optoacoustic, awake, anesthetized animals, resting-state fMRI, system neuroscience

The emergence of awake functional imaging in small animals has revolutionized our ability to examine the intricate relationship between brain activity patterns and specific task components or sequences of stimuli. In contrast to traditional anesthetized imaging, awake imaging provides a more natural and behaviorally relevant context for investigating brain activity. While anesthesia has been widely used in small animal neuroimaging studies to mitigate motion artifacts and physiological stress during functional imaging, it has become apparent that anesthesia can significantly impact brain metabolism, neuronal activity, and neurovascular coupling. Consequently, this limits the translatability of findings to humans (Gao et al., 2017). Moreover, a vast majority of studies in cognitive neuroscience and drug effects necessitate investigations conducted under awake conditions to unravel the underlying neural mechanisms associated with different behaviors (Ferenczi et al., 2016; Behroozi et al., 2020). Fortunately, recent technical advancements have made it possible to immobilize animals during awake imaging while simultaneously examining brain metabolism, neurovascular coupling, and brain circuitry. Consequently, it has now become crucial to advance the field of awake functional imaging in small animals.

Functional imaging techniques can be divided into two main categories: direct and indirect neural activity measurement techniques. Among the indirect neural activity measurement techniques, functional magnetic resonance imaging (fMRI) has emerged as the gold standard for identifying task-dependent local activity patterns by recording hemodynamic responses to neuronal activity through the blood oxygenation level-dependent (BOLD) MRI contrast and most contributions in the current Research Topic deal with this method. Ferris has provided a comprehensive review of the technical requirements and the experimental designs for awake fMRI rodent studies and different applications such as pharmacological MRI, drugs of abuse, sensory evoked stimuli, brain disorders, pain, social behavior, and fear. Over time, fMRI has been applied in various species, including mice, rats, voles, rabbits, cats, dogs, common marmoset monkeys, rhesus macaques, songbirds, and pigeons. In current Research Topic, Weiss et al. reviewed advantages of rabbits over other animal models for awake fMRI studies. Rabbits due to their calmness and ability to remain motionless for several hours significantly reduce possible movement artifacts in fMRI data, making them superior to other small experimental animals.

Complementing fMRI, nuclear imaging tools such as positron emission tomography (PET) provides functional neuroimages using injectable radioactive and biologically active tracers to map brain metabolism and brain perfusion or to monitor neurotransmitter-receptor binding in small laboratory animals. However, investigating transient changes in brain neurotransmitter levels upon behavior, in response to external stimuli or tasks cannot be achieved in anesthetized animals and requires the use of awake and preferentially freely moving animals. In a recent study by Miranda et al., who established imaging studies in awake freely moving and interacting rats in the PET scanner (Miranda et al., 2019), the linear parametric neurotransmitter PET (lp-ntPET) kinetic model was utilized to quantify transient dopamine changes. The authors adapted the spatiotemporal kernel reconstruction for motion correction reconstruction to enable it in awake freely moving rats which resulted in improved lp-ntPET kinetic modeling of noise leading to improved detection of subtle neurotransmitter activations.

Motion artifacts represent a significant confound in awake functional imaging. To mitigate their impact, animals are subjected to body and head restraint during imaging sessions. Although physical restraint is an indispensable technique for reducing motion, it can also induce stress, as can the acoustic noise that accompanies fMRI recordings. These stressors can confound imaging results and compromise their translatability. To address these stresses, Russo et al. implemented a gradual acclimatization procedure during scanning, reducing stress levels and improving imaging quality. However, the current state of research has neglected the investigation of potential sex differences in the habituation response of small animals, although it is widely recognized that male and female rodents exhibit differing responses to stressors (Rincón-Cortés et al., 2019). Lindhardt et al. undertook a study to explore potential sex differences in mice's response to repeated MRI habituation. The researchers monitored various parameters, including heart rate, body weight fluctuations, fecal boli weight, corticosterone concentration levels, the level of the animal's discomfort and the light/dark anxiety test, and this during the MRI habituation session and for both sexes. Their findings revealed that male and female mice exhibited distinctly different responses throughout the habituation protocol and future studies should consider these sex differences when deciding on the habituation procedure.

Awake functional imaging provides a unique opportunity to measure various behaviors and brain responses simultaneously and in real-time but preferentially one should monitor the attentiveness of the animal during its response. Pupil size changes could be reliable markers of brain states and autonomic nervous system activity in response to light, arousal, and cognitive effort. Zeng et al. developed an fMRI-pupillometry platform to study the brain function of awake mice. Real-time pupillometry with fMRI offers a valuable means of monitoring the vigilant states of animals during scanning. For example, it can be used for investigating brain response during different phases of sleep such as rapid eye movement (REM) and Non-REM (Ungurean et al., 2023).

*In vivo* fMRI of small animals provides information at the systems level and could be the intermittent step translating human awake fMRI to its underlying cellular physiology when assisted by awake optical imaging or electrophysiology, be it simultaneous or subsequent in exactly the same imaging space. Mikkelsen et al. developed and optimized an awake-restrained mouse head holder combined with a cranial window that allows for the acquisition of cerebral hemodynamics using MRI and optical imaging tools as laser speckle contrast imaging, optical intrinsic signal imaging and two-photon microscopy during functional activation of the whiskers. Surgical procedures for cranial window and head holder implantation are combined in a preparation suitable for both acute and longitudinal studies.

Recent advancements in technology that have not been covered in the current Research Topic are functional ultrasound (fUS) (Deffieux et al., 2018) and photoacoustic imaging (PAI) (Taruttis and Ntziachristos, 2015) techniques which offer excellent spatiotemporal resolution and allow monitoring freely moving animals via headpost probes ruling out the problem of motion artifacts jeopardizing awake MRI.

In summary, the above investigations published in this Research Topic addressed the challenges and underscored the great potential of awake functional imaging in small animals to improve the translation from preclinical to clinical functional MRI. The field and methods are at a point where open data bases for awake fMRI rodent data start to emerge (Liu et al., 2020) and awake imaging should be used in future study designs whenever possible.

## Author contributions

All authors listed have made a substantial, direct, and intellectual contribution to the work and approved it for publication.
